# *N*,*N*′-Bis(3,3-dimethyl­all­yl)-*N*,*N*′-(prop­ane-1,3-diyl)dibenzene­sulfonamide

**DOI:** 10.1107/S1600536811050562

**Published:** 2011-11-30

**Authors:** Islam Ullah Khan, Tahir Ali Sheikh, Ejaz, William T. A. Harrison

**Affiliations:** a Materials Chemistry Laboratory, Department of Chemistry, GC University, Lahore 54000, Pakistan; b Department of Chemistry, University of Aberdeen, Meston Walk, Aberdeen AB24 3UE, Scotland

## Abstract

In the title compound, C_25_H_34_N_2_O_4_S_2_, the conformation of the linking N—C—C—C—N chain is *gauche*-*anti* [torsion angles = −68.49 (19) and 167.95 (14)°]. The dihedral angle between the aromatic rings is 89.64 (6)°.

## Related literature

For the related structures of *N*-[3-(benzene­sulfonamido)­prop­yl]benzene­sulfonamide and *N*,*N*′-(propane-1,3-di­yl)bis­(*p*-toluene­sulfonamide), see: Sheikh *et al.* (2011 ▶) and Khan *et al.* (2011 ▶), respectively.
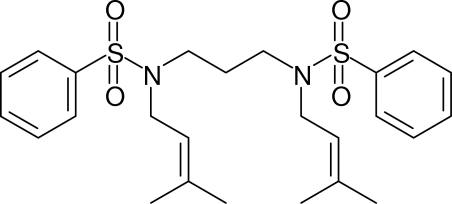


## Experimental

### 

#### Crystal data

C_25_H_34_N_2_O_4_S_2_
*M* *_r_* = 490.66
Monoclinic, 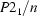

*a* = 10.3019 (2) Å
*b* = 16.3962 (4) Å
*c* = 16.0500 (4) Å
β = 108.449 (1)°
*V* = 2571.71 (10) Å^3^
*Z* = 4
Mo *K*α radiation
μ = 0.24 mm^−1^
*T* = 296 K
0.50 × 0.25 × 0.20 mm

#### Data collection

Bruker APEXII CCD diffractometer
40701 measured reflections
5059 independent reflections
4011 reflections with *I* > 2σ(*I*)
*R*_int_ = 0.028

#### Refinement

*R*[*F* ^2^ > 2σ(*F* ^2^)] = 0.034
*wR*(*F* ^2^) = 0.102
*S* = 1.05
5059 reflections
302 parameters
H-atom parameters constrained
Δρ_max_ = 0.25 e Å^−3^
Δρ_min_ = −0.24 e Å^−3^

### 

Data collection: *APEX2* (Bruker, 2007 ▶); cell refinement: *SAINT* (Bruker, 2007 ▶); data reduction: *SAINT*; program(s) used to solve structure: *SHELXS97* (Sheldrick, 2008 ▶); program(s) used to refine structure: *SHELXL97* (Sheldrick, 2008 ▶); molecular graphics: *ORTEP-3* (Farrugia, 1997 ▶); software used to prepare material for publication: *SHELXL97*.

## Supplementary Material

Crystal structure: contains datablock(s) I, global. DOI: 10.1107/S1600536811050562/zs2161sup1.cif

Structure factors: contains datablock(s) I. DOI: 10.1107/S1600536811050562/zs2161Isup2.hkl

Supplementary material file. DOI: 10.1107/S1600536811050562/zs2161Isup3.cml

Additional supplementary materials:  crystallographic information; 3D view; checkCIF report

## Figures and Tables

**Fig. 1.**
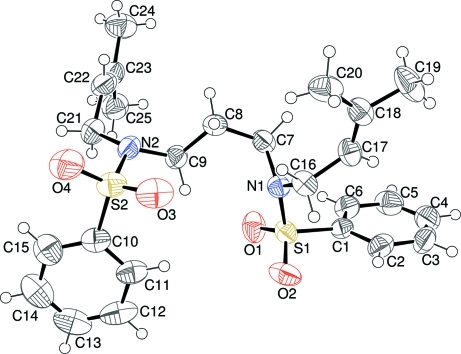
The molecular conformation and atom mumbering scheme for (I) showing 50% probability displacement ellipsoids.

## supplementary crystallographic information

### Comment

As part of our ongoing structural studies of symmetric sulfonamides with a linking propyl chain, including *N*-[3-(benzenesulfonamido)propyl]benzenesulfonamide (II) (Sheikh *et al.*, 2011) and *N*,*N*'-(propane-1,3-diyl)bis(*p*-toluenesulfonamide) (III) (Khan *et al.*, 2011), the synthesis and structure of the title compound, C_25_H_34_N_2_O_4_S_2_ (I), (Fig. 1), are now described.

The dihedral angle between the phenyl rings in the title compound (I) is 89.64 (6)°. The conformations of the atoms of the central chain are *gauche*-*anti* [torsion angles N1—C7—C8—C8 = -68.49 (19)° and C7—C8—C9—N2 = 167.95 (14)°]. The S1—N1—C7—C8 and S2—N2—C9—C8 torsion angles are 116.52 (14)° and -98.69 (16)°, respectively. The bond-angle sums for N1 and N2 are 349.5 and 356.0°, respectively, indicating a significantly greater departure from planarity for N1. In the crystal of (I), only van der Waals' forces are present.

In compound (II) (Sheikh *et al.*, 2011), the conformation of the central N—C—C—C—N chain is *gauche*-*gauche* [torsion angles = 72.5 (5) and 65.7 (5)°]. In compound (III) (Khan *et al.*, 2011), the complete molecule is generated by crystallographic twofold symmetry and the two N—C—C—C fragments have the same *gauche* conformation [torsion angle = 75.53 (14)°].

### Experimental

A mixture of *N*-[3-(benzenesulfonamido)propyl]benzenesulfonamide (0.177 g, 0.5 mmol), sodium hydride (0.24 g; 1.0 mmol) and *N*,*N*-dimethylformamide (10.0 ml) was stirred in a 100 ml round-bottom flask at room temperature for 30 minutes followed by the addition of 3,3-dimethylallyl bromide (0.116 ml; 1.0 mmol). The reaction mixture was stirred for six hours with reaction progress monitored by TLC. At the end of the reaction, the contents were poured over crushed ice. The precipitated product was isolated, washed and recrystallized from methanol to yield colorless blocks.

### Refinement

The hydrogen atoms were placed in calculated positions (C—H = 0.97–0.98 Å) and refined as riding atoms with *U*_iso_(H) = 1.2*U*_eq_(C) or 1.5*U*_eq_(methyl C). The methyl groups were allowed to rotate, but not to tip, to best fit the electron density.

### Crystal data

**Table:**

C_25_H_34_N_2_O_4_S_2_	*F*(000) = 1048
*M**_r_* = 490.66	*D*_x_ = 1.267 Mg m^−^^3^
Monoclinic, *P*2_1_/*n*	Mo *K*α radiation, λ = 0.71073 Å
Hall symbol: -P 2yn	Cell parameters from 4846 reflections
*a* = 10.3019 (2) Å	θ = 2.4–28.3°
*b* = 16.3962 (4) Å	µ = 0.24 mm^−^^1^
*c* = 16.0500 (4) Å	*T* = 296 K
β = 108.449 (1)°	Faceted block, colourless
*V* = 2571.71 (10) Å^3^	0.50 × 0.25 × 0.20 mm
*Z* = 4	

### Data collection

**Table:**

Bruker APEXII CCD diffractometer	4011 reflections with *I* > 2σ(*I*)
Radiation source: fine-focus sealed tube	*R*_int_ = 0.028
graphite	θ_max_ = 26.0°, θ_min_ = 2.4°
ω scans	*h* = −12→12
40701 measured reflections	*k* = −20→20
5059 independent reflections	*l* = −19→19

### Refinement

**Table:**

Refinement on *F*^2^	Primary atom site location: structure-invariant direct methods
Least-squares matrix: full	Secondary atom site location: difference Fourier map
*R*[*F*^2^ > 2σ(*F*^2^)] = 0.034	Hydrogen site location: inferred from neighbouring sites
*wR*(*F*^2^) = 0.102	H-atom parameters constrained
*S* = 1.05	*w* = 1/[σ^2^(*F*_o_^2^) + (0.0458*P*)^2^ + 0.7949*P*] where *P* = (*F*_o_^2^ + 2*F*_c_^2^)/3
5059 reflections	(Δ/σ)_max_ = 0.001
302 parameters	Δρ_max_ = 0.25 e Å^−^^3^
0 restraints	Δρ_min_ = −0.24 e Å^−^^3^

### Special details

**Table:**

Geometry. All e.s.d.'s (except the e.s.d. in the dihedral angle between two l.s. planes) are estimated using the full covariance matrix. The cell e.s.d.'s are taken into account individually in the estimation of e.s.d.'s in distances, angles and torsion angles; correlations between e.s.d.'s in cell parameters are only used when they are defined by crystal symmetry. An approximate (isotropic) treatment of cell e.s.d.'s is used for estimating e.s.d.'s involving l.s. planes.
Refinement. Refinement of *F*^2^ against ALL reflections. The weighted *R*-factor *wR* and goodness of fit *S* are based on *F*^2^, conventional *R*-factors *R* are based on *F*, with *F* set to zero for negative *F*^2^. The threshold expression of *F*^2^ > σ(*F*^2^) is used only for calculating *R*-factors(gt) *etc*. and is not relevant to the choice of reflections for refinement. *R*-factors based on *F*^2^ are statistically about twice as large as those based on *F*, and *R*- factors based on ALL data will be even larger.

### Fractional atomic coordinates and isotropic or equivalent isotropic displacement parameters (Å^2^)

**Table:**

	*x*	*y*	*z*	*U*_iso_*/*U*_eq_	
C1	0.21354 (16)	−0.05844 (10)	0.30778 (10)	0.0423 (4)	
C2	0.1634 (2)	−0.13588 (11)	0.31257 (12)	0.0552 (4)	
H2	0.0878	−0.1434	0.3315	0.066*	
C3	0.2272 (3)	−0.20195 (12)	0.28881 (13)	0.0702 (6)	
H3	0.1948	−0.2543	0.2924	0.084*	
C4	0.3379 (2)	−0.19098 (15)	0.26002 (14)	0.0741 (6)	
H4	0.3803	−0.2358	0.2443	0.089*	
C5	0.3857 (2)	−0.11425 (15)	0.25434 (14)	0.0693 (6)	
H5	0.4603	−0.1070	0.2342	0.083*	
C6	0.32444 (18)	−0.04722 (12)	0.27827 (12)	0.0533 (4)	
H6	0.3575	0.0050	0.2745	0.064*	
C7	0.34337 (16)	0.07689 (10)	0.48435 (11)	0.0475 (4)	
H7A	0.3758	0.0929	0.4362	0.057*	
H7B	0.4104	0.0402	0.5220	0.057*	
C8	0.33084 (19)	0.15195 (11)	0.53634 (11)	0.0520 (4)	
H8A	0.2891	0.1367	0.5804	0.062*	
H8B	0.4216	0.1729	0.5666	0.062*	
C9	0.24648 (18)	0.21859 (10)	0.47923 (11)	0.0455 (4)	
H9A	0.2977	0.2418	0.4437	0.055*	
H9B	0.1634	0.1949	0.4398	0.055*	
C10	−0.06124 (17)	0.30932 (12)	0.43729 (11)	0.0525 (4)	
C11	−0.10347 (19)	0.25338 (14)	0.36942 (12)	0.0602 (5)	
H11	−0.0725	0.1998	0.3773	0.072*	
C12	−0.1930 (2)	0.27902 (17)	0.28927 (13)	0.0721 (6)	
H12	−0.2202	0.2425	0.2426	0.087*	
C13	−0.2414 (2)	0.35669 (18)	0.27828 (16)	0.0784 (7)	
H13	−0.3024	0.3727	0.2245	0.094*	
C14	−0.2010 (2)	0.41129 (17)	0.34560 (17)	0.0815 (7)	
H14	−0.2352	0.4642	0.3377	0.098*	
C15	−0.1093 (2)	0.38832 (14)	0.42576 (14)	0.0684 (5)	
H15	−0.0805	0.4259	0.4713	0.082*	
C16	0.17868 (17)	−0.02590 (10)	0.50808 (11)	0.0466 (4)	
H16A	0.0913	−0.0510	0.4775	0.056*	
H16B	0.1691	0.0026	0.5588	0.056*	
C17	0.28411 (17)	−0.09122 (10)	0.53891 (12)	0.0500 (4)	
H17	0.3022	−0.1228	0.4957	0.060*	
C18	0.35404 (17)	−0.10877 (11)	0.62076 (12)	0.0507 (4)	
C19	0.4535 (2)	−0.17811 (14)	0.64300 (18)	0.0794 (7)	
H19A	0.4279	−0.2157	0.6809	0.119*	
H19B	0.5437	−0.1575	0.6723	0.119*	
H19C	0.4529	−0.2056	0.5901	0.119*	
C20	0.3395 (2)	−0.06355 (14)	0.69819 (13)	0.0698 (6)	
H20A	0.4242	−0.0659	0.7456	0.105*	
H20B	0.2682	−0.0880	0.7164	0.105*	
H20C	0.3167	−0.0077	0.6823	0.105*	
C21	0.26420 (19)	0.36620 (11)	0.52107 (14)	0.0598 (5)	
H21A	0.2230	0.4064	0.5492	0.072*	
H21B	0.2401	0.3800	0.4593	0.072*	
C22	0.41617 (19)	0.36853 (11)	0.56158 (13)	0.0570 (5)	
H22	0.4499	0.3590	0.6217	0.068*	
C23	0.50789 (19)	0.38257 (10)	0.52170 (12)	0.0534 (4)	
C24	0.6577 (2)	0.38660 (15)	0.57265 (16)	0.0778 (6)	
H24A	0.7053	0.3441	0.5531	0.117*	
H24B	0.6701	0.3796	0.6341	0.117*	
H24C	0.6935	0.4387	0.5634	0.117*	
C25	0.4767 (2)	0.39749 (13)	0.42503 (13)	0.0687 (5)	
H25A	0.5287	0.3604	0.4019	0.103*	
H25B	0.5006	0.4525	0.4157	0.103*	
H25C	0.3808	0.3891	0.3957	0.103*	
S1	0.13963 (4)	0.02601 (3)	0.34402 (3)	0.04583 (13)	
S2	0.05674 (5)	0.27959 (3)	0.53939 (3)	0.05475 (14)	
N1	0.21229 (13)	0.03391 (8)	0.44902 (8)	0.0415 (3)	
N2	0.20938 (14)	0.28434 (8)	0.53033 (9)	0.0471 (3)	
O1	0.17552 (15)	0.09743 (8)	0.30526 (8)	0.0640 (4)	
O2	−0.00108 (12)	0.00677 (10)	0.32939 (9)	0.0681 (4)	
O3	0.03302 (15)	0.19581 (9)	0.55383 (9)	0.0731 (4)	
O4	0.04980 (16)	0.33927 (11)	0.60232 (9)	0.0835 (5)	

### Atomic displacement parameters (Å^2^)

**Table:**

	*U*^11^	*U*^22^	*U*^33^	*U*^12^	*U*^13^	*U*^23^
C1	0.0443 (8)	0.0454 (9)	0.0345 (8)	−0.0005 (7)	0.0086 (6)	−0.0038 (7)
C2	0.0622 (11)	0.0518 (11)	0.0466 (10)	−0.0106 (9)	0.0099 (8)	−0.0023 (8)
C3	0.0915 (16)	0.0431 (11)	0.0566 (12)	−0.0016 (10)	−0.0041 (11)	−0.0093 (9)
C4	0.0788 (15)	0.0715 (15)	0.0575 (12)	0.0225 (12)	0.0011 (11)	−0.0207 (11)
C5	0.0568 (11)	0.0875 (17)	0.0642 (13)	0.0109 (11)	0.0197 (10)	−0.0173 (11)
C6	0.0516 (10)	0.0572 (11)	0.0527 (10)	−0.0033 (8)	0.0190 (8)	−0.0081 (8)
C7	0.0428 (9)	0.0455 (9)	0.0535 (10)	0.0049 (7)	0.0141 (7)	0.0040 (8)
C8	0.0537 (10)	0.0494 (10)	0.0440 (9)	0.0010 (8)	0.0031 (8)	0.0001 (8)
C9	0.0548 (10)	0.0393 (9)	0.0406 (9)	−0.0002 (7)	0.0128 (7)	−0.0005 (7)
C10	0.0441 (9)	0.0697 (12)	0.0467 (10)	−0.0065 (8)	0.0187 (8)	0.0031 (9)
C11	0.0517 (10)	0.0731 (13)	0.0529 (11)	−0.0179 (9)	0.0126 (8)	0.0005 (10)
C12	0.0562 (12)	0.1058 (19)	0.0498 (11)	−0.0274 (12)	0.0103 (9)	0.0004 (11)
C13	0.0538 (12)	0.116 (2)	0.0645 (14)	−0.0023 (13)	0.0175 (10)	0.0288 (14)
C14	0.0756 (15)	0.0935 (18)	0.0827 (17)	0.0225 (13)	0.0353 (13)	0.0278 (14)
C15	0.0696 (13)	0.0773 (15)	0.0645 (13)	0.0066 (11)	0.0302 (11)	0.0013 (11)
C16	0.0457 (9)	0.0525 (10)	0.0452 (9)	0.0054 (7)	0.0194 (7)	0.0055 (8)
C17	0.0541 (10)	0.0451 (10)	0.0562 (11)	0.0058 (8)	0.0251 (8)	0.0056 (8)
C18	0.0438 (9)	0.0475 (10)	0.0630 (11)	−0.0008 (7)	0.0202 (8)	0.0158 (8)
C19	0.0620 (12)	0.0681 (14)	0.1120 (18)	0.0155 (10)	0.0332 (12)	0.0454 (13)
C20	0.0659 (12)	0.0840 (15)	0.0531 (11)	−0.0020 (11)	0.0097 (10)	0.0099 (10)
C21	0.0589 (11)	0.0413 (10)	0.0753 (13)	−0.0053 (8)	0.0156 (10)	−0.0067 (9)
C22	0.0615 (11)	0.0498 (11)	0.0531 (11)	−0.0114 (9)	0.0087 (9)	−0.0063 (8)
C23	0.0587 (11)	0.0406 (9)	0.0563 (11)	−0.0075 (8)	0.0118 (9)	−0.0039 (8)
C24	0.0613 (13)	0.0879 (16)	0.0791 (15)	−0.0081 (11)	0.0149 (11)	0.0044 (12)
C25	0.0820 (14)	0.0602 (13)	0.0629 (12)	−0.0052 (10)	0.0213 (11)	0.0020 (10)
S1	0.0477 (2)	0.0487 (3)	0.0404 (2)	0.00946 (18)	0.01282 (18)	0.00255 (18)
S2	0.0538 (3)	0.0726 (3)	0.0396 (2)	−0.0046 (2)	0.01724 (19)	−0.0030 (2)
N1	0.0468 (7)	0.0399 (7)	0.0391 (7)	0.0034 (6)	0.0154 (6)	0.0006 (6)
N2	0.0496 (8)	0.0396 (8)	0.0514 (8)	−0.0048 (6)	0.0150 (6)	−0.0080 (6)
O1	0.0970 (10)	0.0473 (7)	0.0508 (7)	0.0128 (7)	0.0278 (7)	0.0122 (6)
O2	0.0410 (7)	0.0977 (11)	0.0602 (8)	0.0125 (7)	0.0085 (6)	−0.0028 (7)
O3	0.0737 (9)	0.0857 (10)	0.0600 (8)	−0.0195 (8)	0.0212 (7)	0.0211 (7)
O4	0.0794 (10)	0.1212 (13)	0.0523 (8)	0.0133 (9)	0.0240 (7)	−0.0253 (8)

### Geometric parameters (Å, °)

**Table:**

C1—C6	1.381 (2)	C16—N1	1.479 (2)
C1—C2	1.382 (2)	C16—C17	1.494 (2)
C1—S1	1.7651 (16)	C16—H16A	0.9700
C2—C3	1.382 (3)	C16—H16B	0.9700
C2—H2	0.9300	C17—C18	1.315 (2)
C3—C4	1.371 (3)	C17—H17	0.9300
C3—H3	0.9300	C18—C20	1.495 (3)
C4—C5	1.365 (3)	C18—C19	1.496 (3)
C4—H4	0.9300	C19—H19A	0.9600
C5—C6	1.381 (3)	C19—H19B	0.9600
C5—H5	0.9300	C19—H19C	0.9600
C6—H6	0.9300	C20—H20A	0.9600
C7—N1	1.469 (2)	C20—H20B	0.9600
C7—C8	1.515 (2)	C20—H20C	0.9600
C7—H7A	0.9700	C21—N2	1.482 (2)
C7—H7B	0.9700	C21—C22	1.494 (3)
C8—C9	1.512 (2)	C21—H21A	0.9700
C8—H8A	0.9700	C21—H21B	0.9700
C8—H8B	0.9700	C22—C23	1.318 (3)
C9—N2	1.476 (2)	C22—H22	0.9300
C9—H9A	0.9700	C23—C25	1.502 (3)
C9—H9B	0.9700	C23—C24	1.502 (3)
C10—C15	1.378 (3)	C24—H24A	0.9600
C10—C11	1.385 (3)	C24—H24B	0.9600
C10—S2	1.7719 (18)	C24—H24C	0.9600
C11—C12	1.390 (3)	C25—H25A	0.9600
C11—H11	0.9300	C25—H25B	0.9600
C12—C13	1.359 (3)	C25—H25C	0.9600
C12—H12	0.9300	S1—O1	1.4284 (13)
C13—C14	1.363 (4)	S1—O2	1.4284 (13)
C13—H13	0.9300	S1—N1	1.6184 (13)
C14—C15	1.386 (3)	S2—O4	1.4242 (15)
C14—H14	0.9300	S2—O3	1.4270 (15)
C15—H15	0.9300	S2—N2	1.6263 (14)
			
C6—C1—C2	120.38 (16)	C18—C17—C16	126.79 (17)
C6—C1—S1	119.91 (13)	C18—C17—H17	116.6
C2—C1—S1	119.66 (13)	C16—C17—H17	116.6
C3—C2—C1	119.10 (19)	C17—C18—C20	123.69 (17)
C3—C2—H2	120.4	C17—C18—C19	121.47 (19)
C1—C2—H2	120.4	C20—C18—C19	114.83 (18)
C4—C3—C2	120.6 (2)	C18—C19—H19A	109.5
C4—C3—H3	119.7	C18—C19—H19B	109.5
C2—C3—H3	119.7	H19A—C19—H19B	109.5
C5—C4—C3	120.01 (19)	C18—C19—H19C	109.5
C5—C4—H4	120.0	H19A—C19—H19C	109.5
C3—C4—H4	120.0	H19B—C19—H19C	109.5
C4—C5—C6	120.5 (2)	C18—C20—H20A	109.5
C4—C5—H5	119.7	C18—C20—H20B	109.5
C6—C5—H5	119.7	H20A—C20—H20B	109.5
C1—C6—C5	119.36 (18)	C18—C20—H20C	109.5
C1—C6—H6	120.3	H20A—C20—H20C	109.5
C5—C6—H6	120.3	H20B—C20—H20C	109.5
N1—C7—C8	111.98 (13)	N2—C21—C22	111.09 (15)
N1—C7—H7A	109.2	N2—C21—H21A	109.4
C8—C7—H7A	109.2	C22—C21—H21A	109.4
N1—C7—H7B	109.2	N2—C21—H21B	109.4
C8—C7—H7B	109.2	C22—C21—H21B	109.4
H7A—C7—H7B	107.9	H21A—C21—H21B	108.0
C9—C8—C7	112.71 (14)	C23—C22—C21	127.35 (19)
C9—C8—H8A	109.1	C23—C22—H22	116.3
C7—C8—H8A	109.1	C21—C22—H22	116.3
C9—C8—H8B	109.1	C22—C23—C25	125.31 (18)
C7—C8—H8B	109.1	C22—C23—C24	120.94 (19)
H8A—C8—H8B	107.8	C25—C23—C24	113.74 (18)
N2—C9—C8	113.03 (14)	C23—C24—H24A	109.5
N2—C9—H9A	109.0	C23—C24—H24B	109.5
C8—C9—H9A	109.0	H24A—C24—H24B	109.5
N2—C9—H9B	109.0	C23—C24—H24C	109.5
C8—C9—H9B	109.0	H24A—C24—H24C	109.5
H9A—C9—H9B	107.8	H24B—C24—H24C	109.5
C15—C10—C11	120.45 (19)	C23—C25—H25A	109.5
C15—C10—S2	119.67 (15)	C23—C25—H25B	109.5
C11—C10—S2	119.88 (16)	H25A—C25—H25B	109.5
C10—C11—C12	118.6 (2)	C23—C25—H25C	109.5
C10—C11—H11	120.7	H25A—C25—H25C	109.5
C12—C11—H11	120.7	H25B—C25—H25C	109.5
C13—C12—C11	120.8 (2)	O1—S1—O2	119.91 (9)
C13—C12—H12	119.6	O1—S1—N1	106.84 (8)
C11—C12—H12	119.6	O2—S1—N1	107.44 (7)
C12—C13—C14	120.4 (2)	O1—S1—C1	107.54 (8)
C12—C13—H13	119.8	O2—S1—C1	107.08 (8)
C14—C13—H13	119.8	N1—S1—C1	107.48 (7)
C13—C14—C15	120.3 (2)	O4—S2—O3	119.78 (10)
C13—C14—H14	119.8	O4—S2—N2	107.31 (8)
C15—C14—H14	119.8	O3—S2—N2	106.30 (8)
C10—C15—C14	119.4 (2)	O4—S2—C10	107.05 (10)
C10—C15—H15	120.3	O3—S2—C10	108.20 (9)
C14—C15—H15	120.3	N2—S2—C10	107.68 (8)
N1—C16—C17	113.23 (13)	C7—N1—C16	116.35 (13)
N1—C16—H16A	108.9	C7—N1—S1	120.42 (11)
C17—C16—H16A	108.9	C16—N1—S1	119.30 (11)
N1—C16—H16B	108.9	C9—N2—C21	115.59 (14)
C17—C16—H16B	108.9	C9—N2—S2	116.26 (11)
H16A—C16—H16B	107.7	C21—N2—S2	117.54 (12)
			
C6—C1—C2—C3	1.1 (3)	C6—C1—S1—N1	−92.54 (15)
S1—C1—C2—C3	−176.26 (14)	C2—C1—S1—N1	84.80 (15)
C1—C2—C3—C4	−0.7 (3)	C15—C10—S2—O4	17.51 (18)
C2—C3—C4—C5	−0.2 (3)	C11—C10—S2—O4	−163.10 (14)
C3—C4—C5—C6	0.7 (3)	C15—C10—S2—O3	147.88 (15)
C2—C1—C6—C5	−0.6 (3)	C11—C10—S2—O3	−32.73 (16)
S1—C1—C6—C5	176.70 (14)	C15—C10—S2—N2	−97.60 (15)
C4—C5—C6—C1	−0.2 (3)	C11—C10—S2—N2	81.79 (15)
N1—C7—C8—C9	−68.49 (19)	C8—C7—N1—C16	−85.97 (17)
C7—C8—C9—N2	167.95 (14)	C8—C7—N1—S1	116.52 (14)
C15—C10—C11—C12	1.2 (3)	C17—C16—N1—C7	−57.52 (19)
S2—C10—C11—C12	−178.17 (14)	C17—C16—N1—S1	100.26 (15)
C10—C11—C12—C13	−1.9 (3)	O1—S1—N1—C7	−31.34 (14)
C11—C12—C13—C14	1.0 (3)	O2—S1—N1—C7	−161.22 (12)
C12—C13—C14—C15	0.5 (3)	C1—S1—N1—C7	83.85 (13)
C11—C10—C15—C14	0.3 (3)	O1—S1—N1—C16	171.81 (11)
S2—C10—C15—C14	179.66 (15)	O2—S1—N1—C16	41.93 (13)
C13—C14—C15—C10	−1.2 (3)	C1—S1—N1—C16	−73.01 (13)
N1—C16—C17—C18	121.44 (18)	C8—C9—N2—C21	117.43 (17)
C16—C17—C18—C20	−0.9 (3)	C8—C9—N2—S2	−98.69 (16)
C16—C17—C18—C19	177.71 (17)	C22—C21—N2—C9	−68.0 (2)
N2—C21—C22—C23	117.5 (2)	C22—C21—N2—S2	148.57 (14)
C21—C22—C23—C25	−1.5 (3)	O4—S2—N2—C9	170.32 (12)
C21—C22—C23—C24	177.22 (19)	O3—S2—N2—C9	41.04 (14)
C6—C1—S1—O1	22.18 (16)	C10—S2—N2—C9	−74.74 (14)
C2—C1—S1—O1	−160.49 (14)	O4—S2—N2—C21	−46.52 (16)
C6—C1—S1—O2	152.29 (14)	O3—S2—N2—C21	−175.80 (13)
C2—C1—S1—O2	−30.38 (16)	C10—S2—N2—C21	68.42 (15)
